# Crossing the Last Mile of TB Care in Rural Southern Madagascar: A Multistakeholder Initiative

**DOI:** 10.9745/GHSP-D-22-00101

**Published:** 2022-10-31

**Authors:** Nadine Muller, Fierenantsoa Ranjaharinony, Miandrisoa Etrahagnane, Anna Frühauf, Turibio Razafindranaivo, Hortensia Ramasimanana, Julius Valentin Emmrich

**Affiliations:** a Department of Infectious Diseases and Respiratory Medicine, Charité–Universitätsmedizin Berlin, Berlin, Germany.; b Charité Global Health, Charité–Universitätsmedizin Berlin, Berlin, Germany.; c Doctors for Madagascar, Antananarivo, Madagascar.; d Direction Régionale de la Santé Publique, Toliara, Madagascar.; e National Tuberculosis Program, Antananarivo, Madagascar.; f Department of Neurology, Charité–Universitätsmedizin Berlin, Berlin, Germany.; g Berlin Institute of Health at Charité–Universitätsmedizin Berlin, Berlin, Germany.

## Abstract

Despite a free TB care policy, access to TB care in rural Madagascar is limited due to a markedly underfunded health care system. The World Health Organization estimated the yearly TB incidence in Madagascar at 238 cases per 100,000 people in 2020; only half of the patients with TB are being notified and treated. We describe the development, implementation, and lessons learned of an intervention to improve TB care services in a remote, rural district in southern Madagascar. We involved national, regional, and local stakeholders in assessing the multifaceted challenges in a remote, rural area and codesigning activities to address them. The overarching principles of the intervention were to (1) promote national TB guidelines, (2) build on best practices, and (3) prioritize low-cost activities to enable scale-up. An in-depth assessment of challenges in accessing and delivering TB care resulted in the following prioritization of activities: (1) fostering community engagement, (2) decentralizing service provision, (3) improving quality of care, (4) providing nutritional support, and (5) ensuring staff support and supervision. The intervention was launched in September 2019 and is ongoing as of October 2022. During mobile TB clinics conducted between September 2019 and December 2020, 4,982 presumptive patients were screened and 1,706 (34.2%) have been diagnosed with TB. Based on 2010–2020 official TB notification data, we calculated trend-adjusted additional TB notifications during the intervention, resulting in a 2.6-fold increase in cases in 2019–2020. The intervention district’s TB notification rate increased from 178 cases per 100,000 people in 2018 to 424 cases per 100,000 people in 2020. Involving stakeholders from all levels of care was perceived as a key to success. The unexpected increase in the number of patients with TB in the intervention district overburdened the current paper-based TB notification system and emphasized the need for expanded diagnostics and social support services.

## Abstract (precis)

Decentralizing TB care services by offering motorbike-based mobile clinics increased patient accessibility to TB care services in a remote district in Madagascar.

## Teaser

**Key Findings:**

- Bridging gaps along the TB care cascade successfully improved access to TB care and led to a strong increase in TB notifications in the intervention district, one of the most remote and disadvantaged areas of Madagascar.
- Supporting locally active stakeholders in fostering community engagement and decentralizing TB care provision by motorbike-based mobile TB clinics was a key to success.

**Key Implications:**

- Program designers should seek and integrate considerations and good practices from locally experienced health workers when conceptualizing activities to increase the accessibility of TB care.
- To ensure the continuity and quality of TB care, program designers and managers should anticipate measures to cope with shortages in related care and support services, especially if the de facto disease incidence is unclear or estimated to be high.

## INTRODUCTION

Effectively treating TB and containing its spread requires access to diagnostics and continuity of treatment. Yet despite TB being treatable, 3 million people worldwide with active TB remained undiagnosed and untreated in 2019.^1^ The coronavirus disease (COVID-19) pandemic has further reduced access to TB care; in 2020, half of the patients with TB did not receive the essential care they needed.^2^^,^^3^

Low-income countries, in particular, encounter difficulties in effectively closing gaps along the TB care cascade. Especially in rural areas, barriers remain manifold, including poverty, lack of health facilities, health worker shortages, misinformation, corruption, competing traditional healing practices, and social stigma.^4^^–^^8^

In Madagascar, 75% of the population live below the international poverty line of ∼8008 Malagasy Ariary (US$1.90) per day.^9^ Health care access in Madagascar is limited due to an underfunded health care system, resulting in a lack of health care centers and insufficient laboratory facilities for diagnostic services. Six of 10 citizens live in hard-to-reach areas located at least 5 kilometers from the nearest primary health care center with no access other than walking.^10^ With an average of 1.8 medical doctors per 10,000 people, the Malagasy health system is critically short of trained health staff and ranks fifth last in the world in terms of health worker density.^11^ Despite a free TB care policy and considerable commitment to combat TB from national and international stakeholders, the yearly estimated TB incidence in Madagascar has remained largely unchanged since 2013 at 238 cases per 100,000 people.^12^ In 2020, 36,000 new or relapsed TB cases were notified in Madagascar, representing only about half (55%) of the World Health Organization’s estimate of the total TB incidence and indicating an urgent need to improve the access to TB care.^13^ The proportion of HIV coinfection among patients with TB in rural Madagascar is unknown due to a lack of routine HIV testing and the related scarcity of epidemiological data.^14^ It is estimated that around 0.3% of the population aged 15–49 years is living with HIV in Madagascar.^15^

In 2020, only about half of TB cases were being notified and treated in Madagascar, indicating an urgent need to improve the access to TB care.

Established in 1991, Madagascar’s national TB control program conceives, coordinates, and supervises all TB care activities in the country.^16^ The Malagasy TB program is exclusively financed by international donors.^17^ To provide free drug-susceptible TB care, the program relies on a countrywide network of public-sector, faith-based, and private TB diagnostic and treatment centers at the primary care and district levels. Community health workers (CHWs) inform and sensitize communities about TB prevention, symptoms, and treatment and support patient follow-up.

We aimed to improve access to and quality of TB care in Ampanihy, a remote, rural district in southern Madagascar. We share the experiences by describing the development, implementation, and outcome of the intervention. We hope that our findings will help program implementers and policy makers to improve access to TB care in similar settings in sub-Saharan Africa.

## INTERVENTION DESCRIPTION

### Setting

The intervention took place in Ampanihy, a rural district in central Atsimo-Andrefana region with a population of 427,934 in 2020. Atsimo-Andrefana, part of the Malagasy Grand Sud, is the largest and 1 of the poorest regions in Madagascar.^17^ The hard-to-access district of Ampanihy is located approximately 200 kilometers from Toliara, the region’s capital, and can be reached during the dry season in 8–12 hours by offroad vehicle or 16–24 hours by public transport. During the rainy season (December to March), large parts of the district may become inaccessible. Ampanihy is frequently hit by natural disasters such as tropical storms or droughts; in 2021, the district experienced the most severe drought since 1981, resulting in acute food insecurity and a severe humanitarian crisis.^18^^,^^19^

### Stakeholder Mapping and Intervention Design

This intervention was initiated by the nongovernmental organization Doctors for Madagascar in collaboration with local health authorities and the national TB control program in Madagascar. A mapping exercise to identify all stakeholders involved in TB care at the local, regional, and national levels preceded the intervention design. We eventually engaged national and regional TB program leads, public-sector and faith-based TB care providers, community leaders, CHWs, and representatives of local nongovernmental organizations in codesigning the intervention. The participating stakeholders defined the following design principles: (1) promote TB care guidelines as defined by the national TB program, (2) build on best practice experiences from local TB care providers, and (3) prioritize low-cost, high-impact activities to facilitate a potential scale-up to the national level.

### Assessment of Barriers to TB Care and Definition of Activities

We conducted informal group discussions and individual interviews with community members, CHWs, facility-based health workers, and coordinators from the national TB program involved in regional and national program coordination and implementation to understand stakeholders’ challenges in accessing and delivering TB care. We also sought to learn about their experiences, including suggestions on how to overcome those challenges. The Table summarizes the identified barriers by stakeholder group in the intervention district.

**TABLE.** Barriers to Drug-Sensitive TB Care in Ampanihy District, Atsimo-Andrefana Region, Madagascar, 2019–2020

**Type of Stakeholder**	**Type of Barrier**	**Description**
Community representatives	Knowledge	Limited information about free care and/or benefits of TB diagnostics and treatment TB-related fear and stigma
	Geographical	Long distances to health facilities Poor road network, inaccessibility during the rainy season
	Resources	Limited funds for (regular) facility visits, including transportation High opportunity costs for care seeking and regular treatment visits Limited funds for treatment costs of TB-associated complications, which are not covered by the free TB care policy
Community health workers	Knowledge	Limited training opportunities and support structure
	Geographical	Limited mobile network and Internet connectivity hampers communication and data sharing with national TB program Poor road conditions and inaccessibility during the rainy season
	Resources	Lack of motivational incentives Lack of means of transport to visit patients in remote areas Lack of call or data allowance to follow up with patients Lack of comprehensive management and supervision by national TB program
TB care providers	Geographical	Limited mobile network and internet connectivity hampers communication and data sharing with national TB program
	Resources	Frequent supply chain interruptions Insufficient diagnostic equipment and supplies Poor infrastructure, including insufficient space and ventilation, and limited access to permanent electricity or running water Lack of qualified staff Lack of motivational incentives Delays and procedural difficulties in receiving operational budget and communication allowance hampers communication with CHWs and national TB program Lack of communication with national TB program Limited funds and human resources to conduct additional sensitization or follow-up for vulnerable patients Limited funds and human resources to provide social and nutritional support for vulnerable patients Insufficient resources to detect and treat patients with drug-resistant TB
National TB control program coordinators	Geographical	Poor road conditions, inaccessibility during the rainy season
	Resources	Frequent supply chain interruptions Lack of staff for supervising field activities, including for data collection and distribution of operational budgets Delays in obtaining, analyzing, and monitoring TB surveillance data Lack of comprehensive management, monitoring, and communication structures

Abbreviations: CHW, community health worker.

**FIGURE 1:**
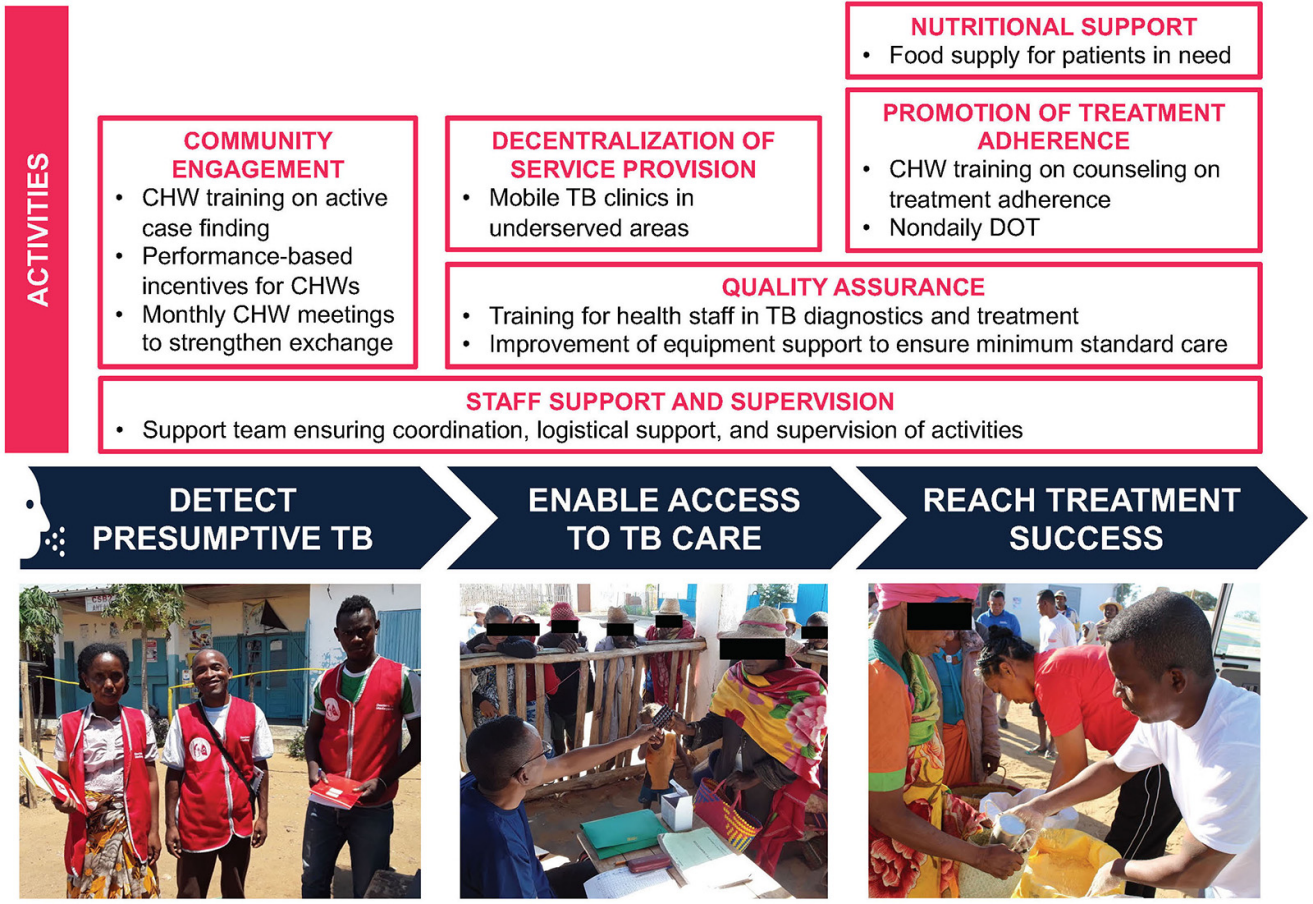
Activities to Improve Drug-Sensitive TB Care in Ampanihy District, Atsimo-Andrefana Region, Madagascar, 2019–2020 Abbreviations: CHW, community health worker; DOT, directly observed therapy.

Based on the assessment of barriers to TB care and stakeholders’ experiences and recommendations, the intervention included the following key components and activities (Figure 1).

#### Community Engagement

To promote health information on TB, 32 CHWs performed group discussions (guided group discussions with community members at risk), mass sensitizations (communication of key messages to the general population over loudspeakers on market days), and home visits. CHWs identified presumptive patients for referral to the mobile TB clinics by using a structured screening tool (including symptoms such as cough for at least 2 weeks, unexplained weight loss, persisting fever, night sweats, bloody sputum, and thoracic pain) as recommended by the national TB program. Although CHWs in Madagascar traditionally fulfill their tasks voluntarily, stakeholders deemed financial incentives essential to increase the performance of CHWs. Therefore, we introduced a low monetary value performance-based bonus scheme rewarding CHWs for referring patients with presumptive TB to the mobile TB clinics.

#### Decentralization of Service Provision

Based on the high impact of geographical barriers on care provision and access, we established motorbike-based mobile TB clinics performed by 4 staff members from 2 district-level TB care facilities. We procured 4 motorbikes, protective clothing and helmets, and equipment for use during mobile TB clinics (tents, tables, chairs, and scales). The TB care staff conducted mobile TB clinics every 2 weeks in each of the 16 villages. The median distance from the TB care facilities to the mobile sites was 42 kilometers (range=18–100 km, 1-way). We scheduled the mobile TB clinics on market days to enable patients from remote areas to reduce their costs. Either CHWs or TB care staff from the mobile TB clinics collected specimens for diagnostic purposes. We implemented a 2-step screening process to increase diagnostic efficiency: (1) CHWs performed a community-based screening, and (2) if deemed presumptive, referred patients to the mobile TB clinics. At the mobile TB clinics, experienced TB health workers took a more detailed history and performed a clinical examination. If confirmed presumptive, further diagnostics were initiated. The diagnosis of pulmonary TB was performed using Ziehl-Neelson sputum smear microscopy. Other diagnostic tools such as chest X-ray, mycobacterial culture, or molecular nucleic acid amplification tests were not routinely available in the intervention district. Depending on travel time, laboratory technicians either processed all samples directly on-site during mobile TB clinics or at the laboratory upon return. In the absence of histopathological testing in the intervention district, the diagnosis of extrapulmonary TB was made based on clinical (i.e., detailed history and clinical examination) and epidemiological (i.e., contact with infectious patients with TB) criteria. TB care staff applied a daily regimen containing 6 months of rifampicin (2HRZE, 4HR) as recommended by national guidelines.

Based on the high impact of geographical barriers on care provision and access, we established motorbike-based mobile TB clinics performed by 4 staff members from 2 district-level TB care facilities.

#### Promotion of Treatment Adherence

All stakeholders perceived daily directly observed treatment schemes to induce high costs and lower treatment adherence. Thus, we implemented an observed treatment every 2 weeks and promoted the relationship between patients and CHWs. The medical visits every 2 weeks included an assessment of treatment side effects, dose adaptation if needed, and a patient’s weight assessment as an indicator of treatment success. The medical visit was deemed mandatory for patients to collect the next 2-week ration of drugs. Patients had to prove the intake of the previous drug ration by handing over empty blisters to health workers.

#### Quality Assurance

We trained 14 TB care staff in basic drug-susceptible TB diagnostics and treatment and 24 health staff and 32 CHWs in health promotion and TB counseling. We provided small medical equipment (e.g., specimen transport boxes, Bunsen burner, timer, weight scales) to TB care facilities based on need. We used health promotion materials from the national TB program to ensure alignment with the national TB strategy.

#### Nutritional Support

We partnered with the World Food Programme to provide food (e.g., rice, soy powder, and vegetable oil) for vulnerable populations including patients with TB. Program staff ensured regular food distribution to patients with TB during mobile TB clinics and at the diagnostic and treatment centers.

#### Staff Support and Supervision

We installed a coordination and support team, consisting of 1 coordinator and 2 intervention staff workers, in the intervention district. Each mobile clinic was accompanied by 1 intervention staff worker who supported TB care staff in performing routine activities and collecting data during mobile TB clinics. The regional coordination team of the national TB program cosupervised all activities by performing clinic visits every 3 to 4 months.

## OUTCOME

We initiated the first mobile TB clinics as a pilot in October–November 2018 and March–June 2019. We launched the intervention sequentially in September 2019 by including CHWs and health staff from the catchment area of 2 of 3 diagnostic and treatment centers in the intervention district, covering approximately 75% of the district’s population.^20^ During the first wave of the COVID-19 pandemic, the intervention was paused for 4 weeks starting March 23, 2020, due to a nationwide lockdown. When activities resumed, we observed changes in patient behaviors regarding the use of TB care services including both promoting factors (i.e., increased attention regarding respiratory symptoms) and inhibiting factors (i.e., fear of infection and associated travel restrictions and social distancing). However, overall use of TB care services did not reduce once activities resumed after the lockdown.

Program monitoring data show that from September 2019 to December 2020, health staff performed 317 mobile TB clinics. Thirty-two CHWs were included in a continuous training program and received performance-based bonuses for active case finding. During mobile TB clinics a total of 4,982 presumptive patients were screened, of which 1,706 (34.2%) were diagnosed with TB, including 16.3% (278/1,706) of cases with extrapulmonary TB. We hypothesize that the overall high positivity rate of 34.2% was due to the 2-step screening process, reducing the number of non-TB patients among the tested patients.

**FIGURE 2:**
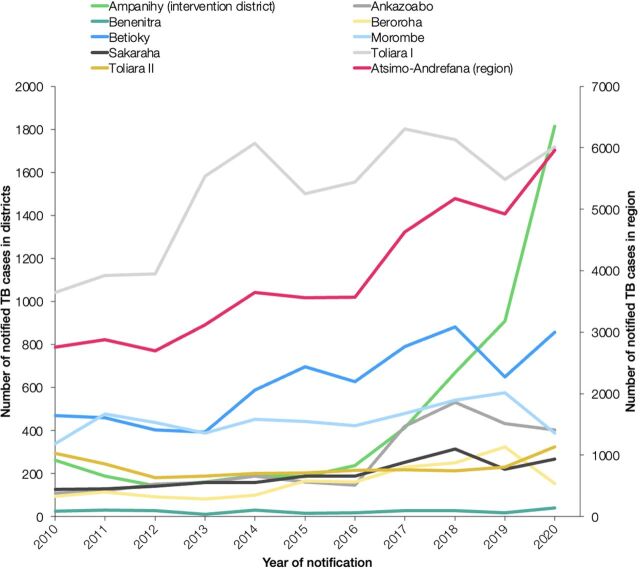
Total Number of Notified TB Cases in Atsimo-Andrefana Region and Districts, Madagascar, 2010–2020

According to official TB notification data, the number of TB cases notified in the intervention district increased from 669 in 2018 to 909 in 2019 and 1,815 cases in 2020 (Figure 2). The proportion of cases with extrapulmonary TB was 38.4% (257/669) in 2018 and decreased to 21.0% (381/1,815) in 2020, most likely due to the increase in cases with pulmonary TB. Based on the official population data (376,052 and 427,934 lived in the intervention district in 2018 and 2020, respectively),^20^^,^^21^ the district’s TB notification rate increased from 178 cases per 100,000 people in 2018 before the intervention to 424 cases per 100,000 people in 2020 during the intervention.

The number of TB cases notified in the intervention district increased from 669 in 2018 to 909 in 2019 and 1,815 cases in 2020.

**FIGURE 3:**
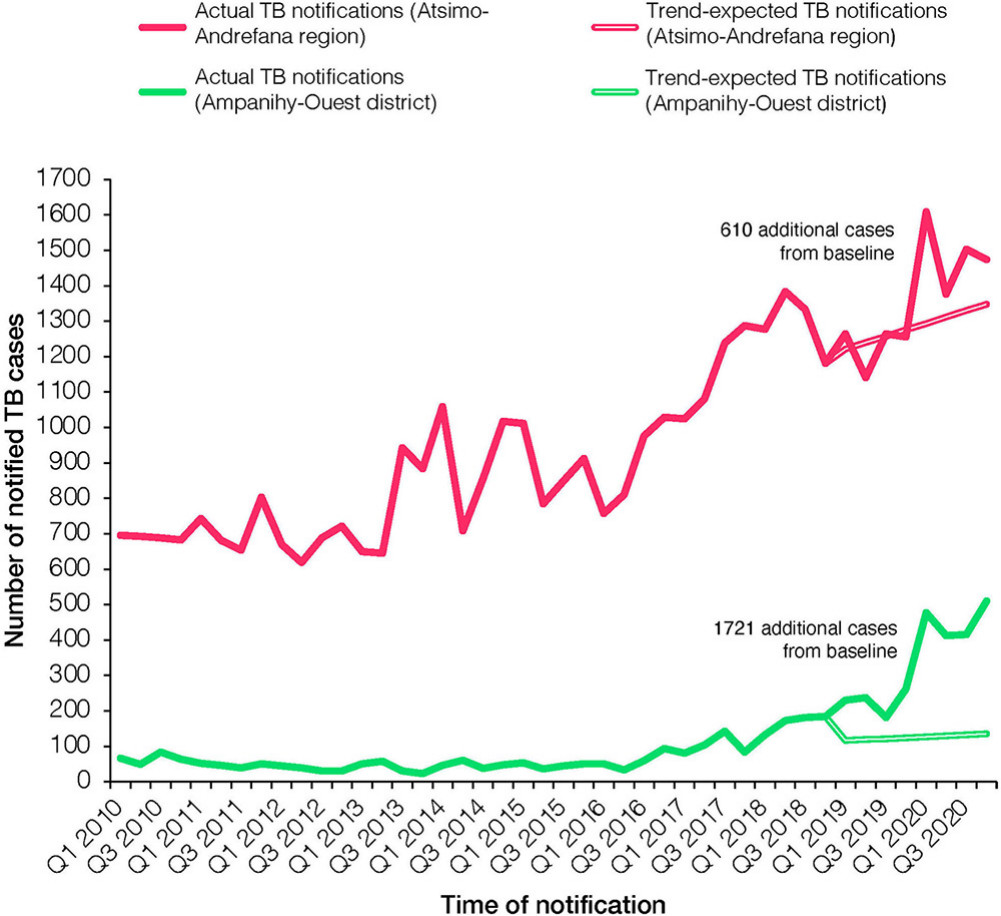
Trend-Expected (Baseline) and Actual TB Notifications in Atsimo-Andrefana Region and Ampanihy District, Madagascar, 2010–2020^a^ ^a^Based on quarterly routine TB surveillance data.

We also calculated the estimated number of additional TB cases in the intervention district and the region during the intervention period following the standard TB REACH methodology.^22^^,^^23^ Based on the official notification data from 2010 and 2018 (before the intervention), we calculated the trend-adjusted number of expected notifications (including smear-positive and smear-negative pulmonary TB, and extrapulmonary TB cases) for 2019 and 2020, the years during which the intervention took place. We then compared the number of expected notifications (baseline) with the number of cases actually notified in 2019 and 2020. The baseline number of trend-expected notifications in the intervention district for 2019 and 2020 was 1,003 whereas 2,724 cases (909 and 1,815 cases in 2019 and 2020, respectively) were notified. Thus, the intervention led to 1,721 additional TB notifications—a 2.6-fold increase (Figure 3).

On the regional level, the total number of notified TB cases in Atsimo-Andrefana region increased from 5,177 in 2018 to 5,962 in 2020 (Figure 2). Using the same analytical approach of trend analysis as previously described, 610 additional cases were notified in Atsimo-Andrefana region in 2019 and 2020, when the intervention took place (Figure 3). The difference in the number of additionally notified cases from baseline (+610 cases on the regional level vs. +1,721 cases on the district level) is likely due to a decrease in the number of notified TB cases in some of the nonintervention districts during the years of the intervention (Figure 2). The number of notified TB cases in districts adjacent to the intervention district (Toliara II, Betioky, and Beninitra) did not decrease in 2019 and 2020; thus, a potential shift of cases from other districts to the intervention district is rather unlikely.

## LESSONS LEARNED

Our intervention resulted in an increase in the number of notified TB cases in the intervention district. The involvement of local, regional, and national stakeholders from different levels of care in the codesign and implementation of the intervention was perceived to be key to success. Some of the activities, notably the decentralization of service provision by motorbike-based mobile TB clinics and specific aspects on how to foster community engagement, were suggested by local health care providers, who drew from past experiences working with an international aid organization. These experiences were 1 of the most important building blocks for the success of the intervention in this complex environment.

The collective experience of local health care providers was an important building block for the success of the intervention.

Two more learnings resulted from the unexpected sharp increase in TB notifications that emerged from the intervention. First, the increase in the number of patients with TB led to an increased demand for specialized services beyond basic drug-susceptible TB care services such as inpatient care for severely affected patients, histopathological diagnostics for the detection of extrapulmonary TB, or treatment options for patients with (presumptive) drug-resistant TB. The high ratio of patients with extrapulmonary TB in the region requires further investigations and more routinely available diagnostic testing. Furthermore, we found that patients with TB faced considerable costs to cover treatment-related expenditures. Thus, the simultaneous implementation of additional social support services may help ensure service provision along the entire TB care cascade. Second, the intervention highly strained the current paper-based TB surveillance and documentation procedures. Health care workers were partially overburdened by the administrative workload, threatening the quality and timeliness of the notification data. The implementation of a context-adapted digital data collection and notification system for TB surveillance (e.g., for use in remote areas with little or no Internet service) was considered a promising future solution to ensure the quality, timeliness, and representativeness of the reported data.

Another learning was related to the rudimentary road conditions in the area, leading to an increased security risk for health care workers performing motorbike-based mobile TB clinics. High-quality protective equipment and rigorous vehicle maintenance turned out to be beneficial for both the security of collaborating partners and trust in the partnership.

### Implications for the National TB Control Program

The high rate of patients with extrapulmonary TB and the lack of routine diagnostic workup is of concern and has prompted further investigation by the national TB program. Currently, the program is collecting individual case data to further characterize this patient group. In addition, a cohort of newly detected extrapulmonary TB cases will undergo extended diagnostic workup including histopathological examination. At present, the existing health infrastructure does not allow for an extended diagnostic workup of all extrapulmonary TB cases.

In 2021, all TB care workers in the region were trained in HIV counseling and testing. Diagnostic tests are now available at TB care facilities and mobile TB clinics. Along with improving the availability of counseling and testing, the destigmatization of HIV among hard-to-reach communities in the region is currently a main priority of the national HIV agenda.

Under the guidance of the national TB program, the intervention is currently being extended to 2 additional rural districts in the Atsimo-Andrefana region to assess TB incidence. In addition, we are conducting a cost-effectiveness study of this intervention to prepare for its expansion to other regions in Madagascar and possible adoption by TB program donors.

Under the guidance of the national TB program, the intervention is currently being extended to 2 additional rural districts in the Atsimo-Andrefana region to assess TB incidence.

## CONCLUSION

Our intervention resulted in a strong increase in the TB notification number in a rural district in southern Madagascar. Community engagement and motorbike-based mobile TB clinics offer a simple and effective solution for reaching rural populations in remote areas. Involving stakeholders from different levels of care in the codesign and implementation of the intervention, including locally experienced health care staff, was key to success. The unexpected increase in the number of patients with TB raised the demand for additional support and emphasized the subsequent need for expanded diagnostics and social services. The shortcomings of the overburdened paper-based TB notification system highlighted the need for a more resilient data collection and surveillance system.

## Notes

**Peer Reviewed**
